# Managing the risks of making the wrong diagnosis: First, do no harm

**DOI:** 10.1016/j.ijid.2021.04.004

**Published:** 2021-05

**Authors:** Piero Olliaro, Els Torreele

**Affiliations:** a Centre for Tropical Medicine and Global Health, Nuffield Department of Medicine, University of Oxford, Oxford, UK; b Institute for Innovation and Public Purpose, University College London, London, UK

**Keywords:** Diagnostics, Misdiagnosis, Dengue, Covid-19

## Abstract

The appropriate use of diagnostics is important as misdiagnosis may have serious consequences. Confidence in a diagnostic test result depends on the test’s accuracy (sensitivity and specificity) in the context of the use-case (who is tested and why) and the prevalence of the condition investigated. Here, we offer an approach to diagnostics focused on the risks and effects of making the wrong diagnosis. We propose ‘fitness brackets’ for a given test to define the range within which the test is fit-for-purpose, based on the use-case and risk-management principles. We use as exemplars tests for dengue pre-vaccination screening and tests for diagnosing Covid-19 in different settings.

Diagnostics are essential public health tools, yet their use, role and complexities are largely misunderstood. Researchers developing a diagnostic test, or end-users choosing among available tests, make choices based on test performance to maximize success in identifying positives and negatives. However, equally important is consideration of the risks and consequences of misdiagnosing someone as having or not having a condition through adopting basic risk-assessment and risk-management reasoning.

Here is why.

First, the essentials of diagnostic tests: performance, deployability and use-case. Performance depends on accuracy: sensitivity—the capacity to identify cases—and specificity—the capacity to exclude non-cases—often at odds with each other, requiring trade-offs depending on the use-case. Deployability depends on performance plus ease of use—from complex, accurate but expensive, low-throughput tests requiring well-equipped laboratories to simpler tests that perform less well but can be done peripherally, with high throughput and short turnaround times. Use-case refers to what questions the test should answer: diagnosing individuals for case management (e.g., identifying whom to treat and monitoring response) or measuring the prevalence of a disease in the population and tracking changes over time (e.g., when deploying an intervention).

While sensitivity and specificity are fixed for any test, the probability that a positive or negative test result is valid varies with the frequency (‘incidence’ or ‘prevalence’) disease occurs in the population tested. Predictive value is expressed numerically as proportions called ‘positive predictive value’ (PPV) and ‘negative predictive value’ (NPV). The PPV is lower when a disease is rare and higher when frequent; the opposite is true for the NPV. PPV is linked to a test’s specificity, while NPV relates to sensitivity, so there are trade-offs between them.

Since confidence in a test result being valid depends on the combination of test accuracy and disease frequency among the individuals tested at that point in time, a practical lens to consider these issues through is the risk of ‘getting it wrong’. What is the probability of the test giving a false-positive (because it is not specific enough) or a false-negative result (not sensitive enough)? These are expressed numerically respectively as 1-PPV (‘false detection rate’ [FDR]) and 1-NPV (‘false omission rate’ [FOR]). Transforming the measurement of success into the measurement of failure is not just a mathematical trick; it means looking at the problem from a different angle and focusing on the risks and consequences of an incorrect diagnosis to help choose which test for which purpose.

For each test with a given accuracy, we can define ‘fitness brackets’ across the range of disease frequency, within which a test is deemed ‘fit-for-purpose’. How these brackets are defined depends on the use-case, the consequences (impact) of false results, and the user risk tolerance for what happens outside the brackets—risks created by false-positives or false-negatives.

To illustrate this concept, consider a test with 90% sensitivity and 95% specificity for a use-case in which the risk tolerance for both false-positive and false-negative rates is 10% (i.e., PPV and NPV are 90%; thus FDR and FOR are 10%) (Figure 1). The fitness brackets range is 33%―50%, i.e., this test is fit-for-purpose with no more than 10% false-positives or false-negatives when the disease prevalence in the population studied is 33%–50%. Below 33%, false-positives increase beyond 10% and increase along the FDR curve as prevalence decreases (e.g., false-positives accounting for 33% of positive results at 10% prevalence, and 51% at 5%―orange dotted lines in Figure 1); above 50% prevalence, there is a growing excess in false-negatives along the FOR curve (e.g., at 75% prevalence 24% of negative results are false-negative―orange dotted lines in Figure 1). In the following text, we apply this to concrete situations to illustrate how thinking about the risks of misdiagnosis can help us navigate these complexities and choose the best test-for-purpose.

**Figure 1:**
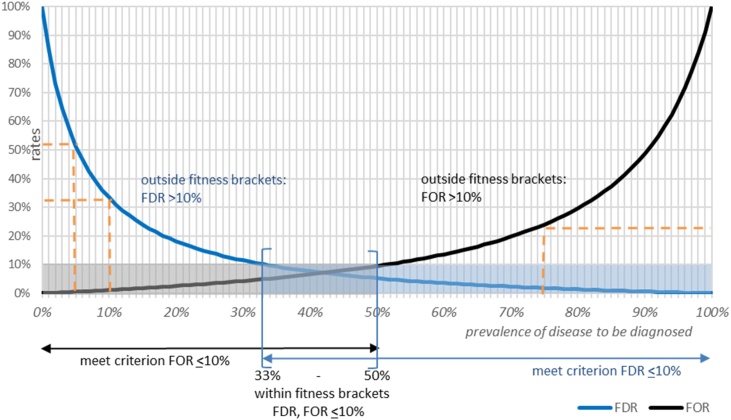
False detection rate (FDR) and false omission rate (FOR) curves for a test with 90% sensitivity and 95% specificity for disease prevalence range of 0%–100%, and fitness brackets when accepting no more than 10% false-positives and 10% false-negatives. FDR is 1-PPV (Positive Predictive Value), where PPV is calculated as = Se ⋅ P/ Se ⋅ P + (1-Sp) ⋅ (1-P); and FOR is 1-NPV (Negative Predictive Value), where NPV is calculated as = Sp ⋅ (1-P) / (1-Se) ⋅ P + Sp ⋅ (1-P). Where Se = Sensitivity; Sp = Specificity; P = Prevalence.

## Dengue pre-vaccination test: putting specificity over sensitivity

**Figure 2:**
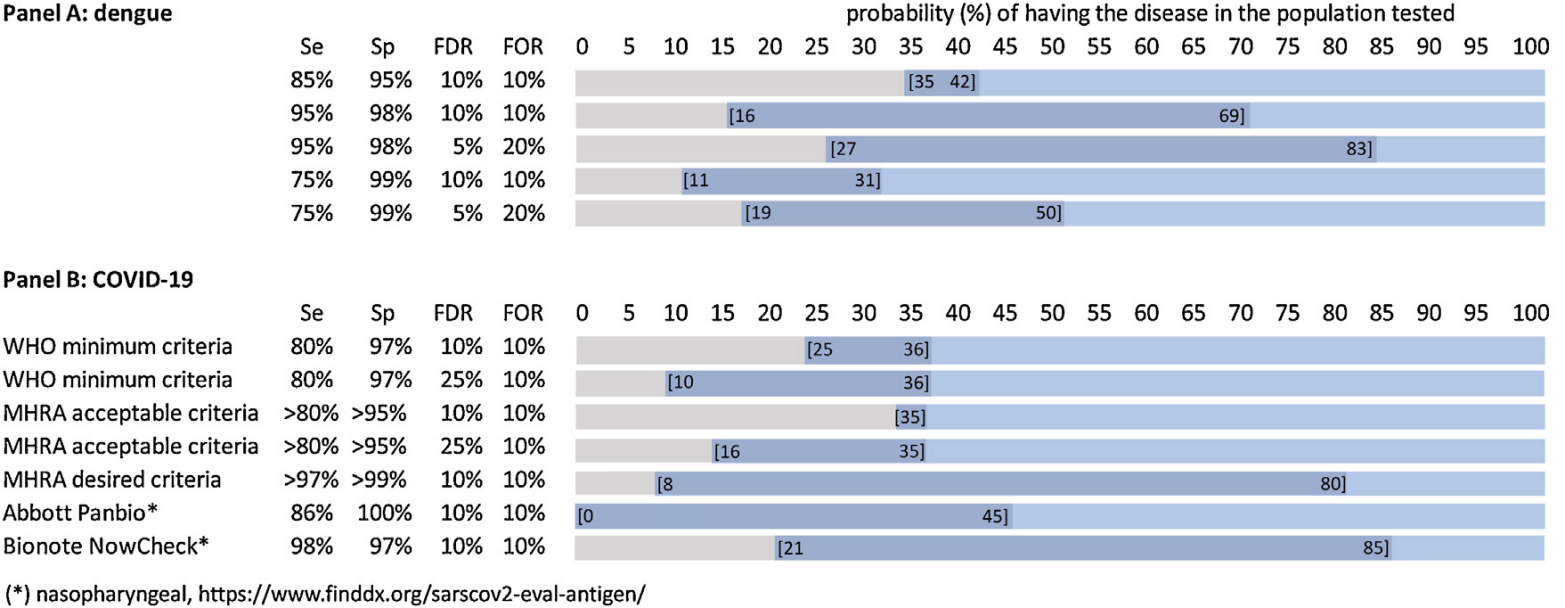
Panel A: Fitness brackets for dengue pre-vaccination screening test; Panel B: Fitness brackets for Covid-19 tests: 1) Test meeting World Health Organisation minimum criteria; 2) Examples from foundatin for innovative new diagnostics (FIND) evaluation of SARS-CoV-2 antigen detecting tests: Abbott Panbio Covid-19 antigen test nasopharyngeal (best specificity); and Bionote NowCheck Covid-19 antigen test nasopharyngeal (best sensitivity/specificity).

(Figure 2A). People living in dengue-endemic areas are infected multiple times during their lives; the second infection may be more severe and potentially life-threatening. The current dengue vaccine carries a risk of severe dengue if given to dengue-naïve individuals, so it should only be given to those with prior dengue virus exposure (Weekly epidemiological record, 2016). In a dengue vaccination programme, all vaccination candidates should first be tested serologically, with an overriding concern not to vaccinate dengue-naïve individuals. To avoid the risk of harming people (and undermining the vaccination programme), a test must be highly specific to avoid false-positives. Meanwhile, health systems also seek to maximise the effectiveness of vaccination programmes at the individual and community level and reduce the risk of excluding subjects who would benefit from a vaccine (false-negatives).

These 2 risks, however, have unequal weights, and thus decision-makers might consider asymmetrical risk-management parameters. This means designing or choosing tests with a preference for specificity over sensitivity and being more concerned with FDR (false-positives) than FOR (false-negatives), especially where dengue prevalence is lower. To safely deliver the vaccine, test specificity as close to 100% is required; sensitivity plays a very minor role. With 99% specificity and accepting the risk that 1/10 positives may be a false-positive (10% FDR), the test is fit-for-purpose when the prevalence of dengue infection in the population to be vaccinated is at least 11%, 10% or 9% when test sensitivity is 75%, 85% or 95%, respectively. If instead, we are more conservative and only accept 1 false-positive in 20 positives (5% FDR), our test will only be suited above 19%, 17% or 16% prevalence when test sensitivity is 75%, 85% or 95%, respectively. Unequal risk tolerance translates into asymmetric fitness brackets that would, for instance, accept false-negative rates of 80% (FOR 20%).

An efficient dengue vaccination programme requires point-of-care rapid diagnostic tests (RDTs) that are cheap and easy to deploy but might be lower performing than laboratory-based methods (DiazGranados et al., 2020). Additional risk-management and minimization measures may therefore be required when prevalence falls outside the test’s fitness brackets. One example would be a 2-step approach, whereby those testing positive on the first screening undergo a second, test with the same or a more specific confirmatory diagnostic. Alternatively, different screening strategies could be applied depending on the background prevalence of infection in the target population.

## Covid-19 testing: different use-cases require different types of tests

(Figure 2B) Active infection (presence of viral RNA or antigen) is detected to identify Covid-19 cases for case management, to reduce transmission (e.g., self-isolation, contact tracing, travel restrictions), or to measure the efficacy of public health interventions (Boehme et al., 2021). Such a wide range of use-cases makes it difficult for any test to meet all the required criteria.

Molecular tests detecting viral RNA via polymerase chain reaction (PCR) are the gold standard. However, they have limited deployability, require laboratory equipment, are relatively expensive, and have limited throughput. Growing numbers of point-of-care antigen-based RDTs (Ag-RDT) are becoming available, though these are generally less sensitive and specific (Test directory - FIND (finddx.org), 2021). The World Health Organization (WHO) recommends that Ag-RDTs be at least 80% sensitive and 97% specific (World Health Organization, 2020).

How will such tests perform? The proportion of swabs testing positive with PCR (considered a proxy for prevalence) has a range of 15%–35% (or more) at the peak of an outbreak wave to 1% in-between waves. If the required FDR and FOR are set at <10%, a test meeting WHO criteria will have narrow fitness brackets between 25%–36% prevalence, meaning it will be fit-for-purpose to detect symptomatic cases in the middle of an outbreak but only narrowly so.

What does risk management outside the fitness brackets of this test entail? Below 25% prevalence, the proportion of false-positives rapidly increases to, for instance, 25%, 55% and 79% at 10%, 3% and 1% prevalence, respectively. A risk-management approach might be to apply asymmetrical brackets and accept a higher risk of false-positives by extending the FDR to, say, 25% (i.e., 1 out of every 4 positive tests could actually be negative), which makes this test fit-for-purpose down to 10% prevalence, if a more specific test is not available.

Depending on screening policies and use-case, prevalence can be <10% even during outbreak waves. Lower still if, for example, screening in-between outbreaks for population-wide cross-sectional surveys or triaging to allow people to travel or go to work or school. These situations are outside the test’s fitness brackets, thus providing unreliable information (unacceptable false-positive rates). The consequences of misdiagnosing would fall on individuals (e.g., inappropriately not allowed to travel, having to self-confine). However, over-diagnosing a condition is a trade-off decision-makers might consider when finding every positive case is deemed essential (sensitivity primes). In situations where prevalence is below the fitness brackets, the risk of false-positives might be mitigated by highly specific Ag-RDTs (example provided in Figure 2B) or a 2-step approach with confirmatory PCR test for each RDT-positive result (Peeling et al., 2021a) or using clinical algorithms.

Above 36% prevalence, the risk is missing cases, i.e., a patient may not receive the correct treatment, will not isolate and might infect others (e.g., 25% false-negatives at 62% prevalence). At such high prevalence, e.g., at the peak of a pandemic wave, the highest sensitivity is required, such as a highly sensitive Ag-RDT (example in Figure 2B) or PCR (World Health Organization, 2020).

For comparison, medicines and healthcare products regulatory agency, UK (MHRA’s) (Anon, 2021) ‘acceptable’ criteria are >80% sensitivity, >95% specificity, and ‘desired’ are >97% and >99%. The former will be fit-for-purpose only at 35% prevalence; allowing greater tolerance for false-positives (e.g., FDR 25%) will extend its use to 16% prevalence. In contrast, a test meeting ‘desired’ criteria will be fit-for-purpose between 8%–80% prevalence, well-adapted to almost all situations.

Risk mitigation includes using and adapting diagnostic algorithms to different tests and risk categories, also accounting for the time in the course of the disease at which the test is conducted (Africa Centres for Disease Control and Prevention (Africa CDC), 2021, Salvatore et al., 2021, Boum et al., 2021, Peeling and Olliaro, 2021).

## Estimating true prevalence

The proposed approach requires an understanding of the true prevalence of the condition of interest. Prevalence may not be known, especially in low-resource settings and in the early stages of an outbreak, and may change over time. One needs not to know the exact prevalence initially but can proceed stepwise to refine the strategy as data accumulate. It is also possible to estimate true prevalence from apparent prevalence obtained with a test of known sensitivity and specificity (Rogan and Gladen, 1978). Bayesian models and online calculators are also available.

Initially, one can define risk categories based on the FDR and FOR slopes, depending on the use-case, and refine as knowledge increases. Dengue vaccination campaigns could be preceded by a cross-sectional survey in the target population to determine seroprevalence level. For Covid-19, while the prevalence varies across different groups, e.g., symptomatic patients, contacts, or asymptomatics, one can start operating on case scenarios within broad prevalence categories as shown in this paper and elsewhere (Peeling et al., 2021b). In both cases, the estimate of true prevalence can be adjusted, allowing for test accuracy using one of the abovementioned methods.

## Conclusions

While diagnostics are commonly judged for their capacity to identify cases from non-cases, the frequency and consequences of misdiagnosis should inform whether a test is fit-for-purpose for which use-case.

The examples provided in this paper illustrate how basic risk analysis steps apply to diagnostic test assessment: identifying the risk; measuring the probability of the risk occurring; quantitating its impact; deciding how to treat it. The specific challenge with diagnostics is that risks pull in opposite directions for false-positive and false-negative results, meaning that one must decide on inherent trade-offs between greater specificity or sensitivity, depending on the use-case and seriousness of risks.

As proposed here, defining ‘fitness brackets’ is an expression of risk acceptance, informing decisions on what test to choose. The brackets can be asymmetrical depending on the significance of the consequences of either under- or over-diagnosing a condition, which will inform the type of risk-mitigation measures. Ideally, this public health level perspective should be applied in the diagnostic test design phase (when defining the target product profile) and certainly when choosing which test to deploy for which purpose from among available tests.

Importantly, to minimise the risks of misdiagnosis, decision-makers should consider diagnostic strategies, such as 2-step testing algorithms, including repeat testing, when prevalence is beyond the inherent limitations of any single test. Such strategies would effectively broaden the range of applicability of testing as a public health intervention.

## Conflict of interest

The authors declare that they have no known competing financial interests or personal relationships that could have appeared to influence the work reported in this paper.

## Funding source

None.

## Ethical approval

Not applicable.

## References

[bib0005] Africa Centres for Disease Control and Prevention (Africa CDC) Interim Guidance on the Use of Rapid Antigen tests for COVID-19 Response. https://africacdc.org/download/interim-guidance-on-the-use-of-rapid-antigen-tests-for-covid-19-response/.

[bib0010] Anon. TPP_Point_of_Care_SARS-CoV-2_Detection_Tests.pdf (publishing.service.gov.uk). 2021.

[bib0015] Boehme C., Hannay E., Sampath R. (2021). SARS-CoV-2 testing for public health use: core principles and considerations for defined use settings. Lancet Glob Health.

[bib0020] Boum Y., Fai K.N., Nicolay B., Mboringong A.B., Bebell L.M., Ndifon M. (2021). Performance and operational feasibility of antigen and antibody rapid diagnostic tests for COVID-19 in symptomatic and asymptomatic patients in Cameroon: a clinical, prospective, diagnostic accuracy study. Lancet Infect Dis.

[bib0025] DiazGranados C.A., Bonaparte M., Wang H., Zhu M., Lustig Y., Schwartz E. (2020). Accuracy and efficacy of pre-dengue vaccination screening for previous dengue infection with five commercially available immunoassays: a retrospective analysis of phase 3 efficacy trials. Lancet Infect Dis.

[bib0030] Peeling R.W., Olliaro P. (2021). Rolling out COVID-19 antigen rapid diagnostic tests: the time is now. Lancet Infect Dis.

[bib0035] Peeling R.W., Olliaro P.L., Boeras D.I., Fongwen N. (2021). Scaling up COVID-19 rapid antigen tests: promises and challenges. Lancet Infect Dis.

[bib0040] Peeling R.W., Olliaro P.L., Boeras D.I., Fongwen N. (2021). Scaling up COVID-19 rapid antigen tests: promises and challenges. Lancet Infect Dis.

[bib0045] Rogan W.J., Gladen B. (1978). Estimating prevalence from the results of a screening test. Am J Epidemiol.

[bib0050] Salvatore P.P., Shah M.M., Ford L. (2021). Quantitative comparison of SARS-CoV-2 nucleic acid amplification test and antigen testing algorithms: a decision analysis simulation model. medRxiv.

[bib0055] Test directory - FIND (finddx.org). [Last accessed 27 February 2021].

[bib0060] Weekly epidemiological record. Dengue vaccine: WHO position paper – July 2016. 29 July 2016, 91^th^ year No 30, 2016, 91, 349–364 http://www.who.int/we.

[bib0065] World Health Organization. Antigen-detection in the diagnosis of SARS-CoV-2 infection using rapid immunoassays. Interim Guidance. 11 September 2020.

